# Psychology service delivery at the Royal Melbourne Hospital Eating Disorder Unit

**DOI:** 10.1186/2050-2974-1-S1-P4

**Published:** 2013-11-14

**Authors:** Megan Dobbie

**Affiliations:** 1 Eating Disorders Unit, NorthWestern Mental Health, Royal Melbourne Hospital, Australia

## 

This poster will outline the psychology services available to inpatients and outpatients who present for treatment at the Royal Melbourne Hospital Eating Disorder Unit. A brief outline of the inpatient, outpatient, and daypatient psychological services will be provided with clear rationales for the basis for offering the service delivery approaches. In addition, the role psychology on the unit as a function of a multipdiscplinary team and therapeutic milleu will be outlined. Future directions for psychology and the role the psychologists working on the unit play in research projects and areas of research interest will also be outlined in this poster.

